# Phospholipases A_2_ (PLA_2_s) and Related Peptides from *Bothrops* Snake Venoms: History, Structure, Pharmacology, and Inhibitors

**DOI:** 10.3390/biom15111583

**Published:** 2025-11-11

**Authors:** Isabela C. dos Santos, Marcela Romanazzi, Geovanna M. Malachias-Pires, Ariani R. Aragão, Eloise T. M. Filardi, Guilherme Melo-dos-Santos, Lara C. Salvador, Marcos F. Cerveja, Anderson M. Rocha, Ananda Magalhães, Isadora S. de Oliveira, José R. Almeida, Norival A. Santos-Filho, Manuela B. Pucca

**Affiliations:** 1 Graduate Program in Bioscience and Biotechnology Applied to Pharmacy, School of Pharmaceutical Sciences, São Paulo State University (UNESP), Araraquara 19060-900, Brazil; isabela.caroline@unesp.br (I.C.d.S.); marcela.romanazzi@unesp.br (M.R.); geovanna.malachias@unesp.br (G.M.M.-P.); e.filardi@unesp.br (E.T.M.F.); guilherme.melo-santos@unesp.br (G.M.-d.-S.); marcos.cerveja@unesp.br (M.F.C.); norival.santos-filho@unesp.br (N.A.S.-F.); 2 Department of Biochemistry and Organic Chemistry, Institute of Chemistry, São Paulo State University, Araraquara 14800-060, Brazillara.cerazi-salvador@unesp.br (L.C.S.); 3 Graduate Program in Tropical Medicine (PPGMT), Amazonas State University (UEA), Manaus 69065-001, Brazil; amr.0609@gmail.com (A.M.R.); anandamglh@gmail.com (A.M.); 4 Department of BioMolecular Sciences, School of Pharmaceutical Sciences of Ribeirão Preto, University of São Paulo, Ribeirão Preto 19040-903, Brazil; 5 Biomolecules Discovery Group, Universidad Regional Amazónica Ikiam, Km 7 Via Muyuna, Tena 150150, Ecuador; rafael.dealmeida@ikiam.edu.ec; 6 School of Pharmacy, University of Reading, Reading RG6 6UB, UK; 7 Department of Clinical Analysis, School of Pharmaceutical Sciences, São Paulo State University, Araraquara 19060-900, Brazil

**Keywords:** *Bothrops*, lancehead pitviper, PLA_2_, PLA_2_ inhibitors, synthetic peptides

## Abstract

Lancehead pitvipers, *Bothrops* snakes, or, popularly, “jararacas”, are common and broadly distributed in the Americas, especially in Brazil, where they are responsible for causing a high number of snakebite accidents. Their venoms are able to induce local and systemic effects, such as hemorrhaging, acute kidney failure, and shock, that can be fatal. Among the compounds of the venom are phospholipases A_2_ (PLA_2_s), which are abundant in some *Bothrops* species. PLA_2_s can perform different activities during envenoming, such as neurotoxicity, myotoxicity, and cytotoxicity, among others, through the hydrolysis of the ester bond at the sn-2 position of phospholipids, producing free fatty acids and lysophospholipids. Although different PLA_2_s can be classified into different PLA_2_ groups and subgroups, according to structure, function, size, localization and Ca^2+^ dependence, they converge to be available in biotechnological and therapeutic applications, such as antiviral and antitumor, among others, being relevant molecules to be deeply studied. Here, we provide the state of the art of PLA_2_s, found in snake venoms, focusing on *Bothrops* venoms, as well as their potential applications, beyond their inhibitors, that also receive attention due their importance in PLA_2_ studies and diverse applications.

## 1. Introduction

**Figure 1:**
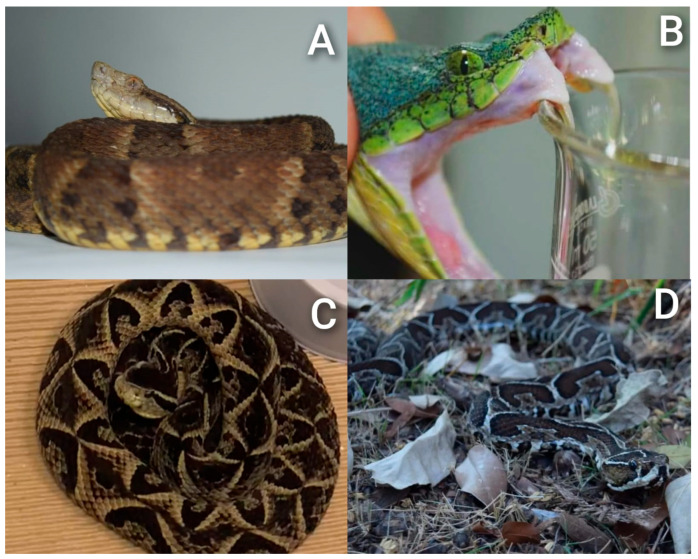
Illustration exemplifying four *Bothrops* species from Brazil. (**A**) *B. atrox* or “Jararaca-da-Amazônia”. (**B**) *B. bilineatus* or “Jararaca-Papagaia”. (**C**) *B. jararacussu* or “Jararacuçu”. (**D**) *B. alternatus* or “Urutu Cruzeiro”. Photo credits: (**A**,**B**) by Anderson Maciel Rocha, (**C**,**D**) by Guilherme Melo-dos-Santos (image archive of the ITox-Lab research group).

*Bothrops* genus is the most diverse within the subfamily Crotalinae, which belongs to Viperidae family. The Viperidae family also includes the genus *Bothrochophias*, which is related to the *Bothrops* group. *Bothrops* are popularly known as “lancehead pitvipers” or “jararacas”, in popular context (Figure 1). With the largest number of species, *Bothrops* has a wide distribution that extends from northeastern Mexico, through Central America, to a broad presence in South America. The genus includes both abundant and widely distributed species, such as *Bothrops asper* and *B. atrox*, as well as rare or restricted species, such as *B. pirajai*, *B. muriciensis* and *B. bilineatus* [1,2,3,4,5,6,7].

Most species are continental, terrestrial, and inhabit forest environments, such as *B. atrox*, *B. jararaca*, and *B. leucurus*. However, some are arboreal in tropical forests (e.g., *B. bilineatus*), and some inhabit open landscapes, such as *B. erythromelas* [6,8,9,10,11,12]. Some species are also found from sea level to elevations as high as 3500 m (e.g., *B. jonathani*, *B. ammodytoides*) [1,2,3,4,5,6]. Currently, there are 63 species of *Bothrops* recorded worldwide, with more than 30 species of *Bothrops* and 2 species of the genus *Bothrochophias* recorded in Brazil [13,14].

**Figure 2:**
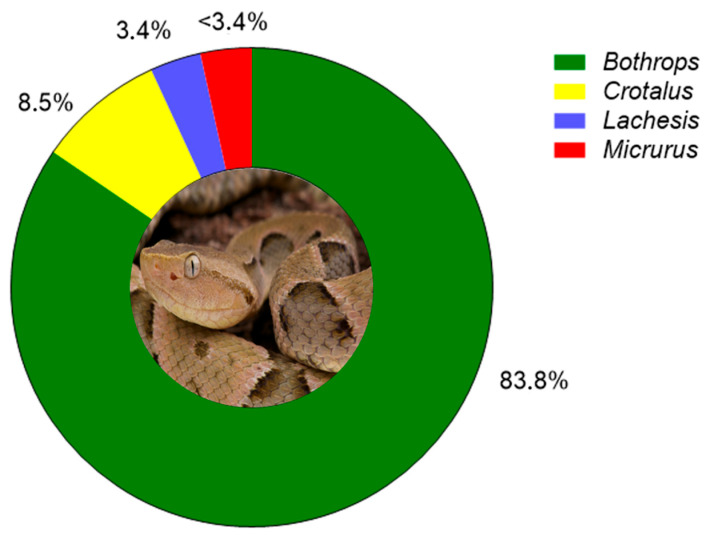
Percentage distribution of snakebite accidents according to snake genus. The central image features *Bothrops jararaca* (popularly known as “jararaca”), the species accountable for the highest incidence of snakebites in Brazil. Among venomous genera, *Bothrops* accounted for 83.8% of cases, *Crotalus* for 8.5%, *Lachesis* for 3.4%, and *Micrurus* for less than 3.4%. The remaining 0.9% correspond to accidents caused by non-venomous snake genera. Adapted from [18,19].

Snakebite accidents are highly neglected and affect millions of people across much of the world. The World Health Organization (WHO) reported that 4.5–5.4 million people are bitten by different snake species each year, resulting in 81–138 thousand deaths, with a number of cases three times greater leading to disabling and permanent sequelae [15,16]. In Brazil, around 30,000 snakebite accidents are reported annually, predominantly caused by *Bothrops* species, followed by those of the *Crotalus*, *Lachesis*, and *Micrurus* genera (Figure 2) [17].

Snakes use venom primarily for immobilizing and digesting their prey [15]. This high incidence is largely due to their widespread distribution across the country (Table 1), and their remarkable ability to adapt to human-altered environments. Different *Bothrops* species are of particular epidemiological importance in various regions of Brazil: *B. atrox* in the Amazon, *B. erythromelas* in the Northeast, *B. jararaca* in the South, Southeast, and Midwest, and *B. neuwiedi* in the Southeast and Midwest [20].

**Figure 3:**
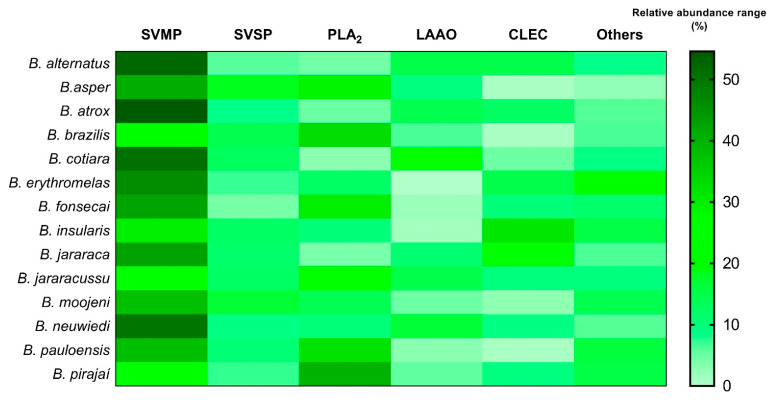
Heatmap comparing the relative abundance range (in percentage) of the components identified in some bothropic venoms from different species. SVMP: snake venom metalloprotease; SVSP: snake venom serine protease; PLA_2_: phospholipase A_2_; CLEC: C-type lectin; LAAO: L-amino acid oxidase; and other minor venom components (disintegrins, vascular endothelial growth factor, peptides, phosphodiesterase, CRISP, nerve growth factor, hyaluronidase, nucleotidase, peptidase, phospholipase inhibitor, glutaminyl cyclase, actin, and undetermined venom components). Adapted from [21,28]. Graphical representations were created using GraphPad Prism version 8.0.1.

Snake venom comprises various constituents, one of which is a complex of proteins. More than 90% of dried venom is made up of proteins, including a wide variety of enzymes, non-enzymatic toxins, and non-toxic proteins [22]. The non-protein fractions include carbohydrates, lipids, biogenic amines, nucleotides, and free amino acids [23,24]. Despite its complexity, the predominant components in snake venom belong to a small number of protein superfamilies. Members of these superfamilies share similar protein structures, although their biological effects can vary significantly [25]. The main components of bothropic snake venoms include phospholipases A_2_ (PLA_2_), snake venom metalloproteinases (SVMPs) and serine proteinases (SVSPs), l-amino acid oxidases (LAOs), nerve growth factor (NGF), C-type lectins (CTLs), and cysteine-rich secretory proteins (CRISP), among others [26,27] (Figure 3).

**Figure 4:**
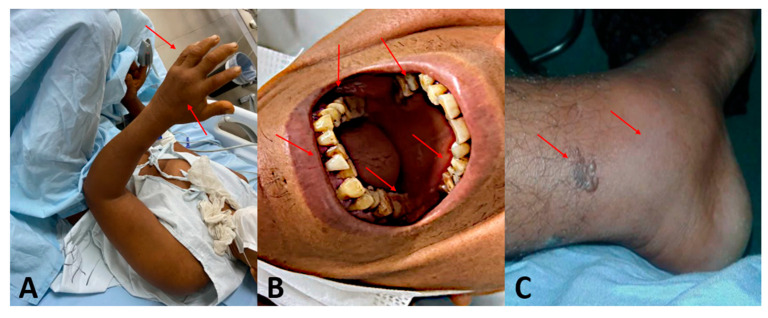
Injuries in patients resulting from snakebite accidents caused by *Bothrops* sp. (**A**) Hand with edema; (**B**) Gum bleeding; (**C**) Ankle with edema and blister formation. Red arrows indicate the affected areas corresponding to the snakebite site or the main local lesions. All images are sourced from the image archive of the ITox-Lab research group. All individuals depicted provided written informed consent for the use and publication of anonymized images, in accordance with institutional ethical guidelines and the approved study protocol (CAAE n. 24120719.5.0000.5302, approved on 24 November 2020).

*Bothrops* venom induces both local and systemic effects (Figure 4) and can be fatal. Local manifestations include bleeding at the bite site, edema, bruising, and pain of varying intensity. Blisters may also develop, containing serous, hemorrhagic, or necrotic fluid. Systemically, the venom is primarily associated with blood incoagulability. In mild to moderate cases, this may present as minor bleeding, such as gingival bleeding, microscopic hematuria, or bleeding from recent wounds. In severe cases, complications can escalate to intense hemorrhaging, acute kidney failure, and shock [29,30,31,32,33].

PLA_2_s are the most abundant proteins in the venoms of different snake genera, exhibiting a wide variety of activities such as neurotoxicity, myotoxicity, cytotoxicity, anticoagulant, hypotensive, cardiotoxic, edema-inducing, and bactericidal properties [34,35,36]. PLA_2_s are widely distributed in nature, being found in a variety of organisms, such as plants, animals (mollusks, arthropods, reptiles, and mammals), fungi, bacteria, and viruses [36,37,38,39,40,41].

Initial publications regarding PLA_2_s from snake venom, by Hanahan et al. (1960) and Doery and Pearson (1961), characterized these enzymes as promoters of the hydrolysis of phosphorylated lipid substrates [42,43]. Subsequently, the discovery of PLA_2_-like proteins (structurally homologous to enzymatically active PLA_2_s but lacking catalytic activity) expanded the functional diversity of this toxin family, resulting in a better understanding of their actions and biological functions [36,37,38,39,40,41].

## 2. PLA_2_: Classification and Mechanistic Pathway

PLA_2_ enzyme belongs to the family of lipolytic enzymes that specifically catalyze the hydrolysis of the ester bond at the sn-2 position of phospholipids, producing free fatty acids and lysophospholipids (LPLs) as the main products [44]. PLA_2_s are amphiphilic and differ from classical water-soluble enzymes in that they act on supramolecular assemblies of phospholipids, such as micelles, vesicles, and liposomes, rather than on soluble substrates. Their catalytic activity is dependent on interaction with the water/lipid interface of these structures, a process referred to as interfacial activation [44,45,46,47].

**Figure 5:**
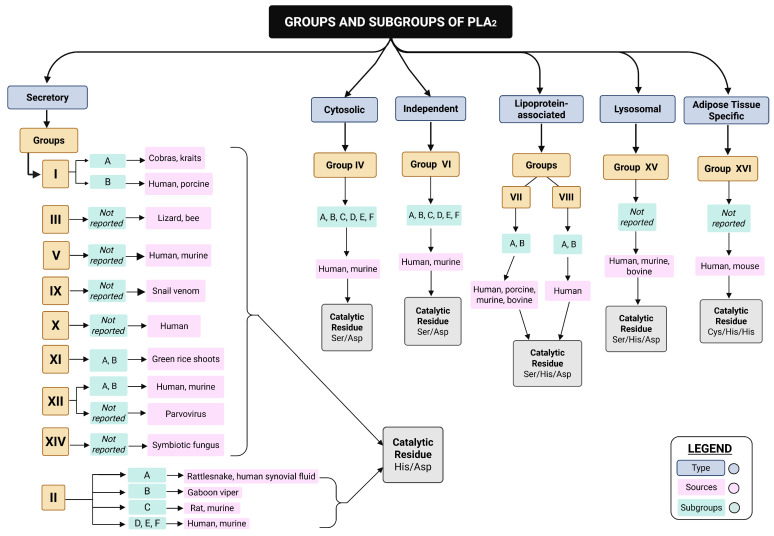
Illustration representing the groups and subgroups of phospholipases A_2_ (PLA_2_) according to their respective alternative names, sources, molecular weights, and catalytic residues. Secretory PLA_2_s are distributed across groups I–XIV, while cytosolic, independent, lipoprotein-associated, lysosomal, and adipose-tissue-specific PLA_2_s are represented by groups IV, VI, VII–VIII, XV, and XVI, respectively. Each group includes distinct subgroups (A–F) and representative biological sources (e.g., snakes, mammals, insects, plants, fungi, viruses). Non-reported subgroups are indicated in the figure, as well as the catalytic residues characteristic of each PLA_2_ class. Not reported: results were not found for this category. Figure created with BioRender.com current release.

The PLA_2_ superfamily is organized into 16 main groups (I to XVI) defined chronologically according to their discovery, which are divided into six subfamilies based on their location in the organism, physiological functions and substrate specificity (Table 2). The six subfamilies are named secreted PLA_2_s (sPLA_2_s) (groups I, II, III, V, IX, X, XI, XII, XIII, and XIV), cytosolic PLA_2_s (cPLA_2_s) (group IV), Ca^2+^-independent PLA_2_s (iPLA_2_s) (group VI), platelet-activating factor acetyl-hydrolase PLA_2_s (PAF-AH PLA_2_s) (groups VII and VIII), lysosomal PLA_2_s (LPLA_2_s) (group XV), and adipose-tissue-specific PLA_2_s (AdPLA_2_s) (group XVI) (Figure 5). Among these subfamilies, sPLA_2_, cPLA_2_, and iPLA_2_ play critical roles in mediating inflammatory processes and the development of neoplasms. In contrast, the PAF-AH PLA_2_, LPLA_2_, and AdPLA_2_ subfamilies are predominantly associated with the progression of conditions such as obesity and atherosclerosis [44,45,47,48,49].

The first three are the most extensively studied [44,50] and the proteins are classified into different groups according to their size, function, sequence, localization, and dependence on the Ca^2+^ ion in their catalytic activity [51].

**Table 2:** Groups and subgroups of PLA_2_s with their respective alternative name, sources, molecular weights and catalytic residues.

Type	Group	Subgroup	Alternative Name	Sources	Catalytic Residue
Secretory	I	A	sPLA_2_	Cobras, kraits	His/Asp
	I	B		Human, porcine	
	II	A		Rattlesnake, human synovial fluid	
	II	B		Gaboon viper	
	II	C		Rat, murine	
	II	D, E, F		Human, murine	
	III	Not reported		Lizard/bee	
	V	Not reported		Human, murine	
	IX	Not reported		Snail venom	
	X	Not reported		Human	
	XI	A, B		Green rice shoots	
	XII	A, B		Human, Murine	
	XII	Not reported		Parvovirus	
	XIV	Not reported		Symbiotic fungus	
Cytosolic	IV	A	cPLA_2_α	Human, murine	Ser/Asp
Cytosolic	IV	B	cPLA_2_β	Human, murine	
Cytosolic	IV	C	cPLA_2_γ	Human, murine	
Cytosolic	IV	D	cPLA_2_δ	Human, murine	
Cytosolic	IV	E	cPLA_2_ε	Human, murine	
Cytosolic	IV	F	cPLA_2_ζ	Human, murine	
Independent	VI	A	iPLA_2_α/iPLA_2_β	Human, murine	
Independent	VI	B	iPLA_2_γ	Human, murine	
Independent	VI	C	iPLA_2_δ	Human, murine	
Independent	VI	D	iPLA_2_ε	Human, murine	
Independent	VI	E	iPLA_2_ζ	Human, murine	
Independent	VI	F	iPLA_2_η	Human, murine	
Lipoprotein-associated	VII	A, B	Lp-PLA_2_/PAF-AH	Human, porcine, murine, bovine	Ser/His/Asp
	VIII	A, B		Human	
Lysosomal	XV	Not reported	LPLA_2_	Human, murine, bovine	
Adipose Tissue Specific	XVI	Not reported	Ad-PLA_2_	Human, mouse	Cys/His/His

Table presenting the groups and subgroups of PLA_2_, characterizing them by type, group, subgroup, alternative names, sources, molecular weights, and catalytic residues. Adapted from [44,52]. Not reported: results were not found for this category.

### 2.1. Secreted PLA_2_ (sPLA_2_)

The first enzymes to be studied and characterized were secreted PLA_2_ (sPLA_2_), with research on snake venoms dating back to 1890 [45]. These enzymes are essential components of the venoms of snakes, scorpions, and other venomous reptiles [45]. sPLA_2_s share common characteristics, such as a reduced molecular mass (13–18 kDa), a high density of disulfide bonds, and a dependence on millimolar concentrations of Ca^2+^ ions for optimal catalytic activity. These enzymes use a histidine residue in the active site and follow a uniform enzymatic mechanism to cleave the sn-2 ester bond of phospholipids [15,52,53]. sPLA_2_ can be classified into two main groups based on their origin: Group I, which includes those found in Old World snakes, species of the Elapidae family; and Group II, which comprises those present in New World snakes, such as jararacas, rattlesnakes, and other species of the Viperidae family. This subfamily has shown continuous growth as new PLA_2_ enzymes are constantly being identified. PLA_2_ enzymes with high sequence homology are grouped within the same class. When more than one homologous PLA_2_ enzyme from the same species is found in venom, each paralog is assigned a subgroup letter, as observed in the IVA, IVB, and IVC PLA_2_ groups [45].

In *Bothrops* snake venoms, PLA_2_s play important roles, such as in the metabolism of structural lipids in cell membranes [36,54]. These two groups share other common characteristics, such as the presence of three long α-helices and two double-stranded β-sheets, which are important for maintaining the stability of the structure [44]. Another similarity is the preservation of the Ca^2+^ binding site, which is important for the performance of the protein’s catalytic function. Meanwhile, Groups I and II of the sPLA_2_s have important characteristics that differentiate them [45]. These include the presence of six conserved disulfide bonds in both groups and a seventh bond formed between residues 11 and 77 in Group I and between residues 50 and 138 in Group II, which contributes to the structural differences between the groups. Another observed difference is the presence of six or seven additional amino acid residues in the C-terminal region of Group II. These residues are negatively charged, influencing the interaction of Group II proteins with the substrate and even with other molecules [44].

Group I sPLA_2_, found in the pancreas of mammals and in the venom of snakes from the Colubridae and Elapidae families, is one of the most widely studied groups and is regarded as a significant model for phospholipase A_2_ enzymology [45]. These enzymes typically contain seven disulfide bridges and molecular masses ranging from 13 to 15 kDa. A distinctive feature of this group is the disulfide bond between the Cys11 and Cys77 residues. In the case of enzymes derived from snake venom, there is a specific structural element known as the elapid loop, which connects the catalytic α-helix to the β-wing. On the other hand, pancreatic PLA_2_s contain an additional segment of five amino acid residues known as the pancreatic loop (residues 62–67). Therefore, these enzymes are divided into subgroups IA and IB, based on the presence of the elapid or pancreatic loop, respectively [15,55,56]. Group IA PLA_2_ enzymes from snake venom possess the elapid loop, which connects the catalytic α-helix to the β-wing [55]. PLA_2_s from subgroup IB, secreted by the pancreas of mammals, are initially released as zymogens. These precursors contain a pro-peptide sequence that is removed by trypsin, enabling enzymatic maturation. In the pancreatic environment, they perform essential functions in the digestion of dietary phospholipids [15,57,58]. The first non-toxic PLA_2_ was identified in bovine pancreatic juice [47].

PLA_2_s from Group II, first identified in rattlesnake and viper venoms and characterized as proteins of approximately 120 amino acids and 14 kDa, contain six conserved disulfide bonds and a seventh between positions 50 and 138, and unlike Group I, Group II lacks the elapid loop and is characterized by a long tail in the C-terminal region of the molecule, where Cys-138 is located [15,47]. Additionally, PLA_2_ enzymes from Group II can be further subdivided based on the amino acid residue present at position 49. Enzymes that have the Aspartic Acid residue at position 49 are called acidic PLA_2_ or Asp49 PLA_2_. This residue can be replaced by Lysine, Serine, Arginine and Asparagine, named Lys49, Ser49, Arg49 and Asn49 basic PLA_2_. These variations lose their catalytic activity, because the substitution of Aspartic Acid at position 49 inhibits the binding of the Ca^2+^ cofactor to the catalytic site loop, but allows the homologous molecule to play another cytotoxic role [15,47,59,60,61]. Of the so-called homologous PLA_2_s, the basic PLA_2_ isoform Lys49 is the most abundant and most commonly studied. Asp49 PLA_2_s and Lys49 PLA_2_-like variants may often coexist in the venom of a given Viperidae snake species, and recent studies demonstrated a synergistic myotoxic effects when in combination [62]. The Ca^2+^ ion plays a crucial role in substrate binding and catalysis by phospholipases, being hepta-coordinated with a pentagonal bipyramidal geometry. Of the seven coordinated interactions, five originate from the protein structure: a bidentate carboxyl group from Asp48 located in a loop of the α-helix and three carbonyl groups (C=O) from the residues Tyr27, Gly29, and Gly31 in the calcium-binding loop. The remaining two interactions involve water molecules, one in the axial region and the other in the equatorial region. The axial water molecule is replaced by the sn-3 phosphate group of the substrate upon binding to the enzyme, while the equatorial water molecule (w5) is polarized and deprotonated by the His47 residue, producing a hydroxide ion that attacks the carbonyl group of the sn-2 ester, forming a tetrahedral intermediate. During this process, His47 becomes protonated via a neighboring water molecule (w6), establishing a proton bridge that reduces the activation energy required for the formation of the intermediate. The decomposition of this tetrahedral intermediate constitutes the rate-limiting step of the enzymatic reaction [15,51,52].

**Figure 6:**
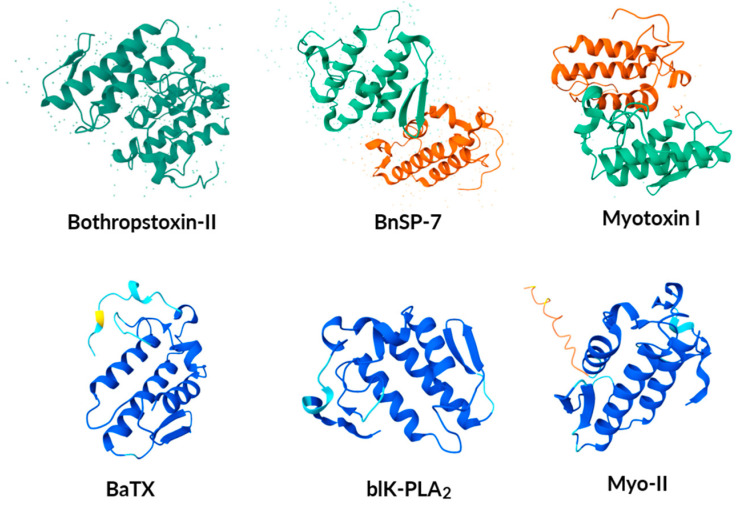
AlphaFold-predicted structures of PLA_2_. Three-dimensional structures of PLA_2_ from different *Bothrops* species predicted by AlphaFold of six different PLA_2_, Bothropstoxin-II (P45881) from *B. jararacussu*, BnSP-7 (Q9IAT9) from *B. pauloensis*, Myotoxin I (P20474) from *B. asper*, BaTX (P86453) from *B. alternatus*, blK-PLA_2_ (P86974) from *B. leucurus* and Myo-II (I2DAL6) from *B. diporus*. The corresponding three-dimensional structures for additional phospholipases can be accessed on UniProt using the identifier numbers listed in Table 3. Graphical representations were created using UniProt AlphaFold Protein Structure Database current release.

The PLA_2_ enzymes from *Bothrops* species, such as Bothropstoxin-II, BnSP-7, Myotoxin I, BaTX, blK-PLA_2_, and Myo-II, belong to the snake venom phospholipase A_2_ (svPLA_2_) group IIA, typical of the Viperidae family. These enzymes share the conserved tertiary structure described for svPLA_2_-IIA, which includes an *N*-terminal α-helix (α1), two disulfide-connected α-helices (α2 and α3) containing the catalytic dyad, an antiparallel β-sheet (β-wing), a Ca^2+^-binding loop, and a flexible C-terminal loop. Unlike the svPLA_2_-IA enzymes from Elapidae that possess the elapid loop, the svPLA_2_-IIA enzymes found in *Bothrops* lack this insertion but feature a C-terminal extension of 5–7 amino acid residues. The highly conserved active site residues (His48, Asp49, Tyr52, Tyr73, and Asp99), as well as the disulfide bonds, maintain the structural integrity essential for catalytic activity and interaction with lipid membranes. This conserved architecture, observed across different snake venom PLA_2_s, including those from *Bothrops*, is crucial for their diverse biological effects, such as myotoxicity and inflammation [63,64] (Figure 6). In addition, they are responsible for mediating severe inflammatory responses induced by the venom’s activity, including edema, pain, and leukocyte migration [65]. These enzymes also exhibit anticoagulant, hemolytic, and necrotic properties, they stand out as targets of interest in the study of toxins and in the development of antivenom treatments [34,41,66,67,68].

**Table 3:** Phospholipases derived from *Bothrops* snake venom.

PLA_2_	*Bothrops* Species	Function	Lenght	UniProt Entry
BaTX	*B. alternatus*	Edema-inducing activities; irreversible neuromuscular blockade.	121 aa	P86453
Myotoxin I	*B. asper*	Local myotoxic activity; anticoagulant action in plasma; edema-inducing; cytotoxic activity; bactericidal activity.	138 aa	P20474
Homolog 2		Lacks enzymatic activity. It is myotoxic and induces a dose-dependent edema in the mouse foot pad. It also exhibits strong anticoagulant effects by binding to factor Xa (F10) and inhibiting the prothrombinase activity. Additionally, it shows cytotoxic activity against a variety of cell types and bactericidal activity against both Gram-negative and Gram-positive bacteria. It also induces a very rapid release of large amounts of potassium ions and ATP from muscle cells.	137 aa	P24605
Braziliase-I	*B. brazili*	Edematogenic activity; mild cytotoxicity on *Trypanosoma cruzi* and *Leishmania infantum*; inhibits ADP- and collagen-induced platelet aggregation.	107 aa	P0DUN3
Braziliase-II			92 aa *	P0DUN4
Myotoxin-I		Exhibits myotoxin and anticoagulant activity; edema-inducing activities; cytotoxic activity against some cell lines and myotubes; antimicrobial activities against *E. coli*, *C. albicans* and *Leishmania*.	78 aa *	P0DQP9
Basic phospholipase A_2_ homolog 2		Myotoxic and displays edema-inducing activities; cytotoxic activity against myotubes	121 aa	P0DTS8
Homolog 2		Lacks enzymatic activity; myotoxic; edema-inducing; cytotoxic activity; antimicrobial activities against *E. coli*, *C. albicans* and *Leishmania*.	121 aa	I6L8L6
Snaclec	*B. diporus*	Interferes with one step of hemostasis	32 aa *	C0HJQ0
sPLA_2_-I		Not reported	138 aa	I2DAL4
sPLA_2_-II			138 aa	I2DAL5
Myo-II			138 aa	I2DAL6
BITP01A	*B. insularis*	Induces edema; produces neuromuscular blockade in chick biventer cervicis; increases CK release and produces myonecrosis; catalyzes the calcium-dependent hydrolysis of the 2-acyl groups in 3-sn-phosphoglycerides.	138 aa	Q8QG87
Acidic phospholipase A_2_		Not reported	107 aa *	P84397
Bothropstoxin-I	*B. jararacussu*	Local myotoxic activity; induces inflammation, edema and leukocytes infiltration; induces NLRP3 NLRP3, ASC (PYCARD), caspase-1 (CASP1), and IL-1beta (IL1B) gene expression in the gastrocnemius muscle, showing that it is able to activate NLRP3 inflammasome also damages artificial and myoblast membranes by a calcium-independent mechanism; bactericidal activity; induces neuromuscular blockade.	137 aa	Q90249
Bothropstoxin-II		Myotoxic activity; induces indirect hemolysis; anticoagulant properties; cytotoxic activities; induces muscle necrosis; polymorphonuclear cell infiltration; edema.	138 aa	P45881
BthA-1		Edema-inducing activities; inhibits phospholipid-dependent collagen; ADP-induced platelet aggregation; anticoagulant activities; bactericidal activity against *E. coli* and *S. aureus*; catalyzes the calcium-dependent hydrolysis of the 2-acyl groups in 3-sn-phosphoglycerides.	138 aa	Q8AXY1
blK-PLA_2_	*B. leucurus*	Myotoxic and edema-inducing activities.	121 aa	P8697
Basic phospholipase A_2_		Cytotoxic; anticoagulant activity; induces Ehrlich tumor growth but not angiogenesis; catalyzes the calcium-dependent hydrolysis of the 2-acyl groups in 3-sn-phosphoglycerides.	122 aa	P86974
Myotoxin II	*B. moojeni*	High myotoxin activities; neurotoxicity; edema-inducing activities; antimicrobial activity against *E. coli* and *C. albicans*; antitumoral activity against some human and mice cell lines.	122 aa	Q9I834
BnpTX-1	*B. pauloensis*	In vitro, shows anticoagulant activity and induces cytotoxicity when tested on C2C12 myoblasts/myotubes. In vivo, when tested on mice, induces myotoxicity (intramuscular injection), edema (injection in the subplantar region) and lethality. Also induces neurotoxic effect on mouse neuromuscular preparations and has bactericidal activity	50 aa *	P0DM51
BnpTX-2		Anticoagulant activity; cytotoxicity when tested on C2C12 myoblasts/myotubes; myotoxicity; edema; catalytic; anticoagulant activities; catalyzes the calcium-dependent hydrolysis of the 2-acyl groups in 3-sn-phosphoglycerides.	35 aa *	P0DM52
BnSP-7		Myotoxic; edema-inducing activity; bactericidal activity; promotes the blockage of the neuromuscular contraction of the chick biventer cervicis muscle; disrupts artificial membranes; tissue damages; edema, necrosis; inflammation; may act as pro-inflammatory mediator.	121 aa	Q9IAT9
Piratoxin-2	*B. pirajai*	Myotoxic activity and edema-inducing activities.	121 aa	P82287

(*): Completed sequence were not stablished yet. Values refer to the partial amino acid sequence. Not reported: results at the UniProt database were not found for this category.

#### Lys49 PLA_2_-like

In 1984, a new type of basic sPLA_2_ was first found in the venom of *Agkistrodon p. piscivorus*, in which the essential Asp49 residue of the catalytic center was replaced by a lysine molecule. By 2023, more than 80 proteins of the same type had been found and characterized in viperid species from the Old and New Worlds, indicating that they form a subgroup of toxins present in many viperid venoms. Initially, the basic isoform of Lys49 was incorrectly classified as having low catalytic activity, but it was later found to be contaminated with low levels of Asp49 sPLA_2_, since both molecules coexist in the same venom. The substitution of the Asp49 residue, together with other modifications, prevents the binding of the Ca^2+^ ion, mainly because the epsilon-amino group of Lys49 is located in the region previously occupied by the ion in Asp49 enzymes. Subsequently, by evaluating Lys49 proteins isolated from other snakes, a toxicity screening revealed the myotoxic activity of PLA_2_-like [69]. This activity was shown to be mainly due to residues located in positions 115–129 of the C-terminal region [70]. Crystallographic, bioinformatic and biophysical studies have shown that dimeric forms are more common in Lys49 PLA_2_ molecules [71]. The “compacted” dimers are highly stable and have been shown to predominate in solution [62], but studies on the importance of the dimeric form of Lys49 PLA_2_-like for the observed myotoxic activity are ongoing [69].

### 2.2. Cytosolic PLA_2_ (cPLA_2_)

Cytosolic PLA_2_s are intracellular enzymes that belong to Group IV of the PLA_2_ family. They are subdivided into six other groups (A–F), which are named cPLA_2_α, cPLA_2_β, cPLA_2_γ, cPLA_2_δ, cPLA_2_ε, and cPLA_2_ζ. The molecular weight of proteins in this class ranges from 85 to 114 kDA, indicating a larger and more complex structure, which is essential for interaction with substrates and performance of enzymatic activity. Unlike sPLA_2_s, Ca^2+^ ions do not contribute to catalytic activity, but bind to two domains that act in these functions: CaLB, formed by eight antiparallel folded β-Sheets, and a catalytic domain composed of 14 β strands and 13 α helices [44].

### 2.3. Ca^2+^ Independent PLA_2_ (iPLA_2_)

The superfamily of Ca^2+^-independent PLA_2_s (iPLA_2_) consists of Group VI enzymes of phospholipases A_2_ (A–F), which are also referred to as iPLA_2_β, iPLA_2_y, and iPLA_2_δ. These enzymes possess molecular weights ranging from 27 to 146 kDA and are present in mammals, as are the two previously mentioned classes. iPLA_2_α are patatins and homologues of patatins found in potato tubers [44]. The structures of iPLA_2_β can be divided into three segments: the *N*-terminal domain, nine ankyrin (ANK) repeats (variations in the iPLA_2_β genome) and the catalytic (CAT) domain. The ANK repeats consist of 33 amino acid residues forming a helix–turn–helix structure followed by a loop. This pattern is only present in two variations, while the others do not have ankyrin repeats. The seventh and eighth ANK repeats form a hydrophobic interaction with the CAT domain, thereby facilitating the formation of a dimer between two iPLA_2_β molecules. This process helps translocation of iPLA_2_β from the cytosol to the membrane and plays a significant role in its catalytic activity [44].

## 3. PLA_2_s Function in Snakebites

Particularly abundant in species of the *Bothrops* genus, PLA_2_s are linked to various biological activities, including acute inflammation, myotoxicity, and nociception. These enzymes degrade phospholipids in cellular membranes, releasing fatty acids such as arachidonic acid, a precursor of key inflammatory mediators like prostaglandins and leukotrienes, which trigger both local and systemic inflammatory responses, contributing to edema and pain observed in snakebite victims. Additionally, PLA_2_s exhibit significant myotoxicity, driven by membrane degradation and necrotic processes, leading to muscle tissue destruction and elevated blood creatine kinase levels, a marker of muscle injury [63,72,73].

The nociception associated with PLA_2_s is another critical aspect, arising from the activation of pain receptors and the release of pro-inflammatory mediators in affected tissues. Beyond their toxic effects, certain PLA_2_ isoforms exhibit unique pharmacological properties, such as anticoagulant activity and the inhibition of platelet aggregation, demonstrating their potential for biomedical applications [74,75].

The PLA_2_ enzymes present in snake venom exert anticoagulant effects, primarily by hydrolyzing plasma phospholipids and disrupting the formation of coagulation complexes [15,76,77,78,79].

This anticoagulant action can occur either through enzymatic hydrolysis of procoagulant phospholipids or via non-enzymatic interactions, where PLA_2_s bind to plasma phospholipids or coagulation factors (e.g., Factor Xa, Factor Va, prothrombin, and thrombin), rendering them unavailable for clot formation [15,80]. These enzymes are also capable of hydrolyzing plasma phospholipids and interacting with factors such as Factor Xa, Factor Va, prothrombin, and thrombin, inhibiting the coagulation cascade [15,81,82]. Thus, the mechanisms by which PLA_2_ enzymes inhibit coagulation are diverse and complex [15].

Anticoagulant PLA_2_ enzymes from snake venom inhibit the formation of the prothrombinase complex, a critical step in coagulation, by binding to factors Xa and/or X. They compete with other factors for the lipid surface or inhibit thrombin [15,82,83]. Interestingly, the anticoagulant potency of these enzymes is not directly related to their enzymatic activity [80]. For example, the acidic PLA_2_ from the Russell’s viper venom (RVVA-PLA_2_-I) inhibits Factor Xa even in the absence of calcium or phospholipids, slowing down the conversion of prothrombin to thrombin [15,76]. The penetrability of PLA_2_, defined as their ability to interact with membranes and hydrolyze phospholipids, is a key determinant of their anticoagulant efficacy [78]. Enzymes with higher penetrability tend to cause greater damage to plasma membranes due to the high density of phospholipids in these structures [84]. Strongly anticoagulant PLA_2_ enzymes exhibit low selective hydrolytic activity, while non-specific ones require higher levels of hydrolysis to exert a significant anticoagulant effect [15,76,85].

Additionally, sPLA_2_ enzymes participate in inflammatory processes by releasing free fatty acids, such as arachidonic acid (AA), which serves as a precursor for pro-inflammatory lipid mediators, including prostaglandins, thromboxane, and leukotrienes. The LPL, a byproduct of hydrolysis, also acts as an inflammatory mediator [86]. sPLA_2_ can release AA through both heparan sulfate proteoglycan (HSPG)-dependent transport mechanisms and direct interaction with the outer leaflet of the plasma membrane. These enzymes hydrolyze phosphatidylcholine to release fatty acids and LPLs, accumulating on the cell surface through interaction with glypicans, which facilitates their internalization and the release of AA [87]. In addition to eicosanoid synthesis, sPLA_2_s stimulate inflammatory cells to produce pro-inflammatory cytokines, independently of their hydrolytic activity, as observed in Groups IB, IIA, V, and X [15,88,89,90].

In the context of neurotoxicity, beta-neurotoxins found in snake venom cause paralysis through complex mechanisms, including the increased pre-synaptic release of AA and Ca^2+^, as well as the activation of PKC, which amplifies acetylcholine (ACh) release and the activity of the protein fusion complex. AA also inhibits the choline uptake transporter, reducing the availability of ACh in the pre-synaptic terminals [15,91,92,93]. These actions contribute to a multifaceted blockade of neuromuscular function, characterized by the desensitization and inactivation of ACh receptors and the depletion of synaptic vesicles. Thus, PLA_2_, through AA, interferes with both pre- and post-synaptic machinery, leading to a prolonged and profound impairment of neuromuscular activity [94]. Due to their broad functional diversity and clinical significance, PLA_2_s remain a focal their therapeutic potential in conditions like chronic inflammation and thrombotic disorders [74,75].

## 4. Review of Phospholipases from *Bothrops* sp. Described in the Literature

As discussed previously, snake venom sPLA_2_s are commonly categorized by their source families: Group I predominantly from Elapidae (including sea snakes within Hydrophiinae) and several Colubridae; Group II from Viperidae, encompassing both Crotalinae and Viperinae. svPLA_2_s isolated from *Bothrops* species belong to Group II, specifically the IIA subclass. Typically, these enzymes have a molecular mass between 13 and 15 kDa, consist of approximately 120 amino acid residues, and are stabilized by 7–8 disulfide bridges. These enzymes exhibit acidic or basic properties depending on the amino acid at position 49, which classifies them as either Asp-49 or Lys-49 types. The Lys-49 type is also referred to as homologous or PLA_2_-like. A conserved catalytic site containing the residues His-48, Asp-49, Tyr-52 and Asp-99 is crucial for phospholipid hydrolysis. Additionally, the calcium-binding loop residues coordinate the catalytic reaction, where Asp-49 plays a critical role in binding calcium. Substituting Asp-49 with Lysine disrupts this interaction, leading to a partial or total loss of catalytic activity [74].

Group IIA phospholipases A_2_ (PLA_2_s GIIA), which have been identified in the venoms of *B. asper*, *B. neuwiedii*, *B. jararacussu*, and *B. insularis*, play a central role in the pathophysiology of envenomation, particularly in triggering inflammatory responses. These enzymes are divided into “classical” Asp49-PLA_2_s, which require Ca^2+^ for catalytic activity, and “variant” Lys49-PLA_2_s, which lack enzymatic function but still cause membrane damage through poorly understood Ca^2+^-independent mechanisms. Both isoforms are known to induce significant local inflammation, characterized by edema, leukocyte infiltration, increased vascular permeability, and the release of inflammatory mediators such as histamine, serotonin, and prostaglandins [72].

The fractionation of venom from *B. atrox* revealed the presence of three significant PLA_2_ fractions, as reported by Sousa et al., 2022 [71]. These fractions showed sequence homologies of 82% with BATXPLA002, and coverage percentages of 94% and 87% with BATXPLA006 and BATXPLA001, respectively. This highlights the structural diversity and specificity of PLA_2_ isoforms within the venom of *B. atrox*, contributing to its complex toxicological profile and diverse biological activities.

Studies conducted by Marinho et al., 2021 [95], with venom from *B. pauloensis* demonstrated that both Asp-49 and Lys-49 PLA_2_ fractions induce significant vascular and functional alterations in isolated kidney systems. The nephrotoxicity caused by these PLA_2_ fractions is associated with oxidative stress mechanisms. Furthermore, both isoforms contributed to toxicity through the release of inflammatory cytokines, highlighting their role in inflammation and organ damage during envenomation. These findings provide insights into the renal effects of *Bothrops* venom components and their underlying mechanisms, which could inform treatment strategies for snakebite-induced nephrotoxicity.

Two basic PLA_2_s, designated PLA_2_-I and PLA_2_-II, were purified from *B. diporus* venom, representing the Asp49 and Lys49 variants, respectively. Both proteins exhibit myotoxicity, cytotoxicity, and the ability to inhibit cell migration. Notably, Lys49 PLA_2_-II proved to be more potent than Asp49 PLA_2_-I in all assays performed. Furthermore, the two proteins act synergistically, amplifying the damage to cultured C2C12 myogenic cells, which highlights their collaborative role in the venom’s toxic effects and the complex tissue damage caused during envenomation [96].

BthTX-II is a basic PLA_2_ isolated from the venom of *B. jararacussu*, as reported by Borges et al., 2021 [97]. Using mass spectrometry, three variants of BthTX-II were identified. The structure of BthTX-IIa is dimeric in its tense state, with a distorted calcium-binding loop. BthTX-IIb, in contrast, is a monomer in the relaxed state, with a fatty acid present in its hydrophobic channel. In an acidic buffer, BthTX-II predominantly exists as a dimer, whereas in a neutral buffer, it adopts a monomeric form. The dimeric assembly is believed to be associated with the non-catalytic myotoxicity of BthTX-II, suggesting that its role in tissue damage occurs independently of its enzymatic activity.

The venom of *B. alternatus* is recognized as a natural source of platelet aggregation inhibitors, with metalloproteinases and phospholipases A_2_ being the primary components responsible for this inhibitory activity. A study by Echeverría et al. (2023) demonstrated that Baltergin, a component of *B. alternatus* venom, inhibits platelet aggregation but lacks the ability to disaggregate pre-formed platelet thrombi [98]. These findings highlight the therapeutic potential of *B. alternatus* venom components in developing novel modulators of platelet function, particularly in conditions where platelet aggregation plays a central role.

Research conducted in the UniProt database for “*Bothrops* phospholipases” identified 43 results. Analyzing the provided results, we identified 24 phospholipases (Table 3), 9 phospholipase inhibitors (see item 7), 3 metalloproteinase, 1 serine proteinase, 1 type-C lectin and other 5 phospholipases derived from different snake genus and species that were once *Bothrops* sp.

## 5. Biotechnological and Therapeutic Applications of PLA_2_s and Peptides Derived from Snake Venom PLA_2_s

PLA_2_s from snake venoms are extensively studied enzymes that have gained prominence in the scientific community due to their broad spectrum of associated biotechnological activities [99]. In addition to their numerous characteristics from both biological and structural perspectives, the range of pharmacological activities associated with these enzymes is of significant medical and scientific interest because of their relationship with various human diseases [100]. The adverse effects caused by these enzymes, such as inflammation, cytotoxicity, myotoxicity, neurotoxicity, and hypotension, have become attractive targets for biotechnological and therapeutic research, as PLA_2_s are considered prime candidates for therapeutic drug targets [101,102,103]. Scientific studies worldwide aim to understand the potential applications of PLA_2_s from different snake species. Currently, the roles of these enzymes in inducing antitumor, anti-angiogenic, anti-inflammatory, antimicrobial, antiparasitic, hypotensive, antithrombotic, anticoagulant, pharmacological, clinical, and industrial activities are already well documented [36,100,102,103,104,105].

The table below (Table 4) demonstrates the potential biotechnological and therapeutic applications of PLA_2_s from the venoms of different snake species worldwide, correlating the categories with the potential applications described in the literature and the corresponding bibliographic references.

As previously mentioned, animal venoms are a mixture of complex and bioactive molecules, such as proteins and peptides, with biologically interesting properties that can be studied for the development of new drugs [118]. A notable example is Captopril, one of the most widely used antihypertensives in the world for treating hypertension. It is derived from a bradykinin-potentiating peptide (BPP) found in the venom of *B. jararaca*. Captopril was the first drug derived from animal toxins to be approved by the FDA (Food and Drug Administration) in 1981 [119,120].

Based on the primary structure of PLA_2_s, several cationic peptides have been synthesized and have demonstrated antimicrobial, antitumor, and antiparasitic efficacy, showing significant pharmacological interest [121]. The literature provides numerous examples of peptides derived from the C-terminal region of PLA_2_s.

The Mt-II peptide, derived from the terminal region of the myotoxin Mt-II from *B. asper* (residues 115–129 KKYRYYLKPLCKK), has demonstrated a variety of biological activities, including antitumor effects and antibacterial potential similar to that of the parental molecule *in vitro*. This activity occurs through a mechanism involving membrane permeabilization [122,123]. To enhance the peptide’s antimicrobial activity and reduce its toxicity to eukaryotic cells, various modifications to its amino acid sequence were tested. Among these, the analog named pEM-2 (sequence KKWRWWLKALAKK) stood out, showing greater potency and specificity against prokaryotes and lower toxicity to eukaryotic cells [122]. Building on this, another study analyzed the antitumor activity of the pEM-2 analog (D-enantiomer), which demonstrated satisfactory inhibitory effects on EMT6 mammary tumors in mice [124].

Derived from the C-terminal region of bothropstoxin I from *B. jararacussu*, the peptide p-BthTX-I (residues 115–129 KKYRYHLKPFCKK) demonstrated antimicrobial activity against Gram-positive and Gram-negative bacteria, including multidrug-resistant strains, while showing no activity against *Candida albicans*, indicating specificity [125,126]. To optimize the activity and production of the peptide, modifications were made to its amino acid sequence, resulting in the dimeric peptide [des-Cys11, Lys12, Lys13-(p-BthTX-I)_2_K], named (p-BthTX-I)_2_K. This modified peptide exhibited enhanced antimicrobial activity and low toxicity to eukaryotic cells [126]. Furthermore, the molecule and its analogs showed antiviral activity against SARS-CoV-2, the causative agent of COVID-19, combined with low cytotoxicity and promising selectivity indices [127].

**Figure 7:**
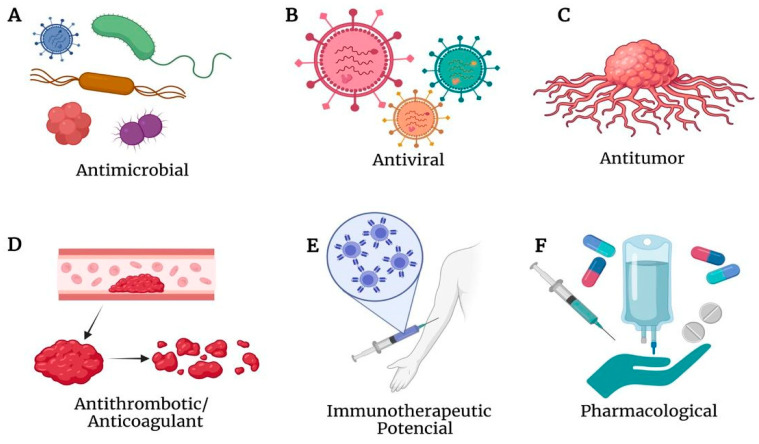
Illustration demonstrating the spectrum of selected biotechnological and therapeutic applications of PLA_2_s. (**A**) Antimicrobial potential (Mt-II from *B. asper;* p-BthTX-I and (p-BthTX-I)_2_K from *B. jararacussu*; MTX-I and MTX-II from *B. brazili*). (**B**) Antiviral effect ((p-BthTX-I)_2_K from *B. jararacussu*). (**C**) Antitumoral effect (Mt-II and pEM-2 from *B. asper)*. (**D**) Antithrombotic and anticoagulant agent. (**E**) Immunotherapeutic potential. (**F**) Pharmacological potential in the discovery of new drugs and medicines as phospholipases are considered prime candidates for therapeutic drug targets. Figure created with BioRender.com current release.

Costa and collaborators (2008) evaluated the cytotoxic effect of synthetic peptides derived from the C-terminal region of myotoxins MTX-I, an Asp49 PLA_2_, and MTX-II, a Lys49 PLA_2_, from the venom of *B. brazili*. The peptides pepMTX-I (residues 115–129 RKYMAYLRVLCKK) and pepMTX-II (residues 115–129 KKYRYHLKPLCKK) demonstrated antimicrobial activity against *E. coli* and *C. albicans*, as well as antiparasitic activity against *Leishmania* sp. and cytotoxicity against JURKAT cell lines [106]. Studies indicate that synthetic peptides derived from PLA_2_s in snake venoms of the *Bothrops* genus are promising alternatives for biotechnological applications. These peptides are smaller, easy to obtain, and capable of mimicking the biological activities of the parental molecules [128]. In general, the future prospects for the biotechnological and therapeutic applications of PLA_2_s (Figure 7) from snake venoms and synthetic peptides derived from PLA_2_s are encouraging. They hold potential for developing new therapies using these peptides to treat various diseases, as well as for improving and advancing methods and technologies based on recent discoveries.

## 6. Phospholipase Inhibitors

As previously described in this study, phospholipases constitute a vast group of enzymes with diverse biological functions. In this section, we focus exclusively on phospholipase A_2_ inhibitors derived from snake venoms. Similarly, the class of PLA_2_ inhibitors is equally extensive. As of the date of this article, a search for the term “*Phospholipase inhibitors*” in the UniProt database yields approximately 2000 sequences related to this enzyme class. Also in this database, when searching for *Bothrops*-derived PLAs, we could identify gamma-type secreted phospholipase (Y PLA) inhibitors also derived from bothropic venom. Most PLA_2_ inhibitors do not have a described function in the scientific literature. One of the few with a described function is the myotoxin inhibitor protein, which binds directly to phospholipase A_2_ in the presence or absence of calcium. It exhibits anti-enzymatic, anti-myotoxic, anti-edema, anti-cytotoxic, anti-bactericidal, and anti-lethal properties against both basic and acidic phospholipases A_2_ from *Bothrops* venoms [129] (Table 5).

PLA_2_ enzymes, particularly those from *B. jararaca*, have been extensively studied, along with their inhibitors. The development of PLA_2_ inhibitors as potential anti-inflammatory agents has garnered significant attention. This interest stems from the role of PLA_2_ in the release of arachidonic acid from membrane phospholipids, a rate-limiting step in eicosanoid production. Beyond eicosanoid synthesis, PLA_2_-mediated hydrolysis of membrane phospholipids also initiates the generation of platelet-activating factor (PAF), a potent inflammatory mediator. Therefore, the inhibition of PLA_2_ activity presents a theoretically robust strategy for anti-inflammatory intervention [52,130].

The existence of multiple PLA_2_ isoforms complicates the elucidation of cellular mechanisms that regulate AA release and the subsequent production of eicosanoids. Due to factors influencing PLA_2_ kinetics and the uncertainty surrounding which PLA_2_ isoform(s) regulate AA release—potentially involving more than one—it has been challenging to design or isolate specific inhibitors. A brief review of the literature indicates that most PLA_2_ inhibitors exhibit topical anti-inflammatory activity. This may be attributed to the hydrophobic nature of these inhibitors, which facilitates their absorption through the skin. However, when administered orally, their absorption appears to be limited, potentially reducing their efficacy in systemic applications [131].

In the pursuit of phospholipase inhibitors (PLIs) for diverse applications, such as modulating inflammatory responses and enhancing the neutralization of snake venoms, considerable attention has been directed towards identifying these inhibitors in snakes themselves. This approach stems from the unique immunity of snakes to their own venom, which provides a natural model for resistance. PLIs isolated from snake blood exhibit significant variability among species and are commonly characterized as oligomeric, globular, acidic glycoproteins with molecular weights ranging from 75 to 180 kDa. These distinct molecular features suggest their potential as specialized tools for biochemical and therapeutic applications [132,133,134].

**Figure 8:**
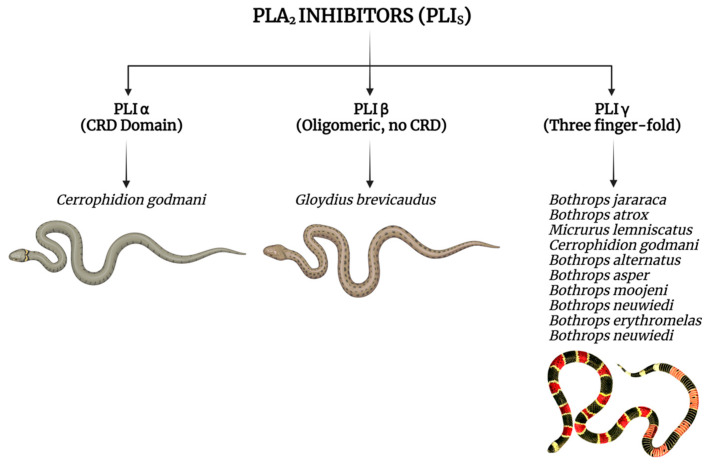
Classification of PLI types based on snake species. Figure created with BioRender.com current release.

The mechanism of action of secreted PLA_2_ inhibitors relies on the formation of a soluble complex between the inhibitor and the target enzyme, effectively neutralizing the enzyme’s activity. Structurally, these inhibitors are classified into three groups—α, β, and γ (Figure 8)—based on their unique characteristics. Remarkably, a single snake species, whether venomous or non-venomous, may possess inhibitors from all three groups, underscoring the evolutionary diversity and adaptability of these molecules. Inhibitors from these groups have been successfully isolated from a wide range of snake species across different families, further emphasizing their broad distribution and potential relevance in nature. These findings highlight the utility of snake-derived PLIs as promising candidates for the development of therapeutic agents aimed at controlling PLA_2_-mediated pathological processes, including inflammation and envenomation [134,135].

PLIβ is a PLA_2_-inhibitory protein present in the serum of venomous snakes, playing a crucial role in neutralizing toxic PLA_2_s that may leak into the circulation. Purified from the serum of the Chinese mamushi snake *Gloydius brevicaudus* (formerly *Agkistrodon blomhoffii siniticus*), PLIβ contains nine tandem leucine-rich repeats (LRRs) and shares 33% sequence identity with human leucine-rich α2-glycoprotein (LRG), whose function remains unclear. Specifically, PLIβ inhibits group II basic PLA_2_ from *G. brevicaudus* venom, without affecting the acidic or neutral forms of the enzyme. Additionally, PLIβs with similar inhibitory spectra have been found in the serum of non-venomous Colubridae snakes, such as *Elaphe quadrivirgata* and *Elaphe climacophora*, suggesting a conserved role for this inhibitor across different species. The discovery that PLIβ can tightly bind cytochrome c (Cyt c), a pro-inflammatory and danger-associated molecule, suggests a broader protective function beyond PLA_2_ inhibition, potentially contributing to regulation of inflammatory processes [136].

PLIβ is a PLA_2_-inhibitory protein found in the blood of snakes, specifically serving as an ortholog of leucine-rich α2 glycoprotein (LRG). This protein plays a crucial role in neutralizing the toxic effects of PLA_2_ from the snake’s own venom. In this study, snake LRGs were purified from various snake serum samples using Cyt c affinity chromatography, and it was found that all purified LRGs were dimers linked by disulfide bonds. Among the different snake species analyzed, *Elaphe climacophora* PLIβ exhibited weaker inhibitory activity against *Gloydius brevicauda* basic PLA_2_ than the PLIs from *G. brevicauda* and *Elaphe quadrivirgata*, both of which are known to neutralize *G. brevicauda* basic PLA_2_ effectively. Notably, *P. flavoviridis* LRG showed no inhibitory activity against basic venom PLA_2_, further emphasizing the variation in the inhibitory abilities of PLIβ across different species. Despite its potential to inhibit PLA_2_, the actual functionality of snake LRG as a PLA_2_-inhibitory protein (PLIβ) is species-dependent, and its role remains variable among different snakes [137].

Estevão-Costa et al. (2008) conducted an investigation into the presence of γ-PLIs in six *Bothrops* species: *B. alternatus*, *B. erythromelas*, *B. jararaca*, *B. jararacussu*, *B. moojeni*, and *B. neuwiedi* [138]. They found γ-PLI transcripts in all species analyzed, with the mature proteins composed of 181 amino acid residues, including a 19-residue signal peptide. While the proteins from *B. erythromelas* and *B. neuwiedi* differed from the others, the study concluded with the identification of six new γ-PLIs in Brazilian *Bothrops* species. From a therapeutic perspective, the oligopeptide QPFPGLPLSRPNGYY, observed in this study, stands out as a promising candidate for the development of new PLA_2_ inhibitors.

In the study conducted by Picelle et al. (2017), the researchers analyzed PLIγs from snake species across various families by aligning amino acid sequences and constructing a phylogenetic tree [134]. The study confirmed the presence of PLIγ inhibitors in *B. atrox*, showing significant similarity to other known PLIγ sequences. Sequences from *M. lemniscatus* demonstrated specific similarities with elapid and crotaline snakes at conserved sites. Notably, the three-finger fold characteristic of PLIγs, coupled with the absence of a CRD domain specific to PLIαs, and a tertiary structure similar to CNF, strongly suggest that the inhibitors in question belong to the PLIγ class. This research deepens the understanding of the evolutionary relationships between these inhibitors and their functional roles in snake venom.

In the venom of *Cerrophidion godmani* (formerly *Bothrops godmani*), two myotoxin inhibitors, CgMIP-I and CgMIP-II, were directly isolated from the snake’s blood plasma through selective binding to affinity columns containing myotoxins I or II, respectively. Both proteins are glycosylated, acidic (pI = 4), and composed of 20–25 kDa subunits forming 110 kDa (CgMIP-I) or 180 kDa (CgMIP-II) oligomers. In inhibition assays, CgMIP-I specifically neutralized the PLA_2_, myotoxic, edema-forming, and cytolytic activities of myotoxin I, while CgMIP-II selectively inhibited the toxic properties of myotoxin II. *N*-terminal amino acid sequencing and cDNA analysis revealed that CgMIP-I resembles γ-type inhibitors, sharing a cysteine residue pattern from the Ly-6 protein superfamily, while CgMIP-II shares sequence identity with α-type inhibitors, containing carbohydrate recognition domains similar to those found in C-type lectins and mammalian PLA_2_ receptors. These findings highlight the structural and functional diversity of these inhibitors and their potential pharmacological applications [132].

Recent studies have focused on endogenous snake inhibitors, as highlighted by Fernandes et al. (2024), who assessed the inhibition of the A2 fraction of PLA_2_ from *B. jararaca* venom using extracts from five *Siparuna* species [139]. These plants have a long-standing history in traditional medicine for the treatment of snakebites in tropical regions. The extracts showed significant inhibition of the venom’s proteolytic activity (30–96%) and plasma coagulation (75–800s), as well as up to 90% inhibition of PLA_2_ activity. Moreover, five alkaloids identified within the extracts were found to possess strong antivenom potential, highlighting the therapeutic promise of *Siparuna* extracts in neutralizing venomous effects.

## 7. Conclusions

PLA_2_s from *Bothrops* snake venoms represent a complex group of enzymes with remarkable structural diversity and significant implications for human health. Their well-documented roles in envenomation, particularly in mediating inflammatory, myotoxic, neurotoxic, and anticoagulant effects, highlight their pathophysiological relevance and the need for continued research on their mechanisms of action. The increasing understanding of their structural variants, such as the Asp49 and Lys49 isoforms, has clarified their synergistic roles in tissue damage and inflammation. Furthermore, the biotechnological and therapeutic potential of PLA_2_s and their synthetic derivatives is evident in their demonstrated antimicrobial, antiviral, antitumor, and anti-inflammatory properties, as supported by experimental and clinical evidence. The identification of natural and synthetic PLA_2_ inhibitors, including those derived from *Bothrops* species themselves, adds another promising dimension for developing novel therapeutic strategies to mitigate PLA_2_-mediated damage and inflammation. Taken together, the comprehensive characterization and continued exploration of snake venom PLA_2_s and their inhibitors may not only improve antivenom therapies but also foster the development of new bioactive compounds with potential applications beyond toxinology.

## 8. Methodology

This review was conducted between November 2024 and July 2025 and aimed to comprehensively analyze the structural, functional, and biological diversity of snake venom phospholipases A_2_ (PLA_2_). A systematic literature search was performed across multiple scientific databases, including PubMed, SciELO, and NaBi, using combinations of keywords such as phospholipase A_2_, snake venom, toxin structure, venomics, and inhibitors. Relevant original research articles, reviews, and proteomic studies published were included. Protein sequences and structural data were retrieved from the UniProt database (https://www.uniprot.org), and three-dimensional structural models were accessed through the AlphaFold Protein Structure Database (https://alphafold.ebi.ac.uk/) via UniProt. Data visualization and figure generation were performed using GraphPad Prism version 8.0.1., GraphPad Software, San Diego, CA, USA) for statistical representation and plotting. Illustrations and schematic diagrams were created with BioRender (https://biorender.com) to support the visualization of molecular mechanisms and structural features. All data collected from these platforms were analyzed qualitatively, and results were organized into thematic categories, including structural diversity, enzymatic mechanisms, biological activities, and therapeutic potential of PLA_2_ and PLA_2_-like proteins.

## Figures and Tables

**Table 1:** Geographic distribution of *Bothrops* snakes. Global and Brazilian geographic distribution of *Bothrops* snake species.

*Bothrops* Species	Country	Brazilian Region	Brazilian State
*B. alcatraz*	Brazil	Southeast	SP
*B. alternatus*	Argentina, Brazil, and Uruguay	South, Southeast, and Midwest	MS, GO, MG, RJ, SP, PR, SC, and RS
*B. ammodytoides*	Argentina	Na.	Na.
*B. asper*	Belize, Colombia, Costa Rica, Ecuador, Guatemala, Honduras, Nicaragua, Mexico, Panama, Peru, and Venezuela	Na.	Na.
*B. atrox*	Bolivia, Brazil, Colombia, Ecuador, English Guiana, French Guiana, Peru, Suriname, Trinidad, and Venezuela	North, Midwest, and Northeast	RR, AP, AC, RO, AM, PA, TO, MT, and MA
*B. ayerbei*	Colombia	Na.	Na.
*B. barnetti*	Peru	Na.	Na.
*B. bilineatus*	Bolivia, Brazil, Colombia, Ecuador, English Guiana, French Guiana, and Suriname	North, Northeast, Southeast, and Midwest	RR, AP, RO, AM, PA, MT, CE, PE, AL, BA, ES, MG, and RJ
*B. brazili*	Bolivia, Brazil, Colombia, English Guiana, French Guiana, Peru, Venezuela, and Suriname	North, Northeast, and Midwest	AC, RO, AM, PA, MT, and MA
*B. caribbaeus*	Saint Lucia and Antilles	Na.	Na.
*B. chloromelas*	Peru	Na.	Na.
*B. cotiara*	Argentina and Brazil	Southeast and South	SP, PR, SC, and RS
*B. diporus*	Argentina, Bolivia, Brazil, and Paraguay	Southeast and South	SP, PR, SC, and RS
*B. erythromelas*	Brazil	Northeast and Southeast	PI, CE, RN, PB, PE, AL, SE, and BA
*B. fonsecai*	Brazil	Na.	MG, RJ, and SP
*B. germanoi*	Brazil	Southeast	SP
*B. insularis*	Brazil	Southeast	SP
*B. itapetiningae*	Brazil	Midwest, Southeast, and South	MS, GO, DF, MG, SP, and PR
*B. jabrensis*	Brazil	Northeast	PB
*B. jararaca*	Argentina, Brazil, and Paraguay	Midwest, Southeast, and South	GO, BA, ES, MG, RJ, SP, PR, SC, and RS
*B. jararacussu*	Argentina, Bolivia, Brazil, and Paraguay	Northeast, Southeast, and South	BA, ES, MG, RJ, SP, PR, SC, RS, and MS
*B. jonathani*	Argentina and Bolivia	Na.	Na.
*B. lanceolatus*	Antilles	Na.	Na.
*B. leucurus*	Brazil	Northeast and Southeast	CE, RN, PB, PE, AL, SE, BA, ES, and MG
*B. lutzi*	Brazil	North, Midwest, and Southeast	TO, GO, DF, MA, PI, CE, PE, BA, and MG
*B. marajoensis*	Brazil	North and Northeast	AP, PA, and MA
*B. marmoratus*	Brazil	North, Midwest, and Southeast	TO, GO, DF, and MG
*B. mattogrossensis*	Bolivia and Brazil	North, Midwest, and Southeast	RO, AM, TO, MT, MS, GO, and SP
*B. medusa*	Venezuela	Na.	Na.
*B. monsignifer*	Bolivia	Na.	Na.
*B. moojeni*	Argentina, Bolivia, Brazil, and Paraguay	North, Midwest, Southeast, and Northeast	TO, MT, MS, GO, DF, MA, PI, BA, MG, SP, and PR
*B. muriciensis*	Brazil	Northeast	AL
*B. neuwiedi*	Argentina and Brazil	Midwest, Southeast, and South	GO, DF, BA, MG, RJ, SP PR, SC, and RS
*B. oligobalius*	Bolivia, Brazil, Colombia, English Guiana, French Guiana, Peru, Venezuela, and Suriname	North	AP, AM, and PA
*B. oligolepis*	Peru	Na.	Na.
*B. osbornei*	Ecuador and Peru	Na.	Na.
*B. otavioi*	Brazil	Southeast	SP
*B. pauloensis*	Bolivia and Brazil	Midwest and Southeast	MT, MS, GO, DF, MG, SP, and PR
*B. pictus*	Peru	Na.	Na.
*B. pirajai*	Brazil	Northeast	BA
*B. pubescens*	Brazil and Uruguay	South	SC and RS
*B. pulcher*	Colombia and Ecuador	Na.	Na.
*B. punctatus*	Colombia, Ecuador, and Panama	Na.	Na.
*B. sanctaecrucis*	Bolivia	Na.	Na.
*B. sazimai*	Brazil	Southeast	ES
*B. smaragdinus*	Bolivia, Brazil, Colombia, Ecuador, English Guiana, French Guiana, and Suriname	North	AC, RO, and AM
*B. sonene*	Peru	Na.	Na.
*B. taeniatus*	Bolivia, Brazil, Colombia, Ecuador, English Guiana, French Guiana, and Peru	North, Midwest, and Northeast	RR, AP, AC, RO, AM, PA, MT, and MA
*B. venezuelensis*	Venezuela	Na.	Na.

*Bothrops* snake’s species that can be found worldwide including specific considerations of the Brazilian territory. RR—Roraima, AP—Amapá, AC—Acre, RO—Rondônia, AM—Amazonas, PA—Pará, TO—Tocantins, MT—Mato Grosso, MS—Mato Grosso do Sul, GO—Goiás, DF—Distrito Federal, MA—Maranhão, PI—Piauí, CE—Ceará, RN—Rio Grande do Norte, PB—Paraíba, PE—Pernambuco, AL—Alagoas, SE—Sergipe, BA—Bahia, ES—Espírito Santo, MG—Minas Gerais, RJ—Rio de Janeiro, SP—São Paulo, PR—Paraná, SC—Santa Catarina, RS—Rio Grande do Sul. Adapted from [21]. Na.: not applicable.

**Table 4:** Potential biotechnological and therapeutic applications of phospholipases A_2_ (PLA_2_s) from snake venoms, organized by application category.

Application Category	Description	Ref.
Antimicrobial	Activity against pathogenic bacteria, fungi, and protozoa; cytotoxic activity.	[106,107]
Antiviral	Potent virucidal (neutralizing) activity against SARS-CoV-2; potential HIV inhibitor by blocking host cell invasion.	[108,109]
Antitumor	Inhibition of tumor cell adhesion and migration; inhibition of angiogenesis.	[99,110,111]
Anti-inflammatory	Modulation of the inflammatory response, reduction/inhibition of cytokines and inflammatory mediators.	[55,112]
Antithrombotic/Anticoagulant	Prevention of platelet aggregation and blood clot formation.	[82,113]
Hypotensive	Vasodilation and blood pressure reduction.	[114]
Immunotherapeutic Potential	Low immunogenicity suggests potential immunosuppressive effects.	[115,116]
Industrial Applications	Use in bioremediation processes, formulation of antivenom serums, and specific inhibitors.	[105,117]
Pharmacological	PLA_2_ enzymes, by binding to target proteins, may induce their pharmacological effects.	[36,105]

**Table 5:** PLA_2_ inhibitors derived from bothropic venom.

PLA_2_ Inhibitor	Snake	Lenght	Uniprot Entry
Myotoxin inhibitor protein	*B. moojeni*	166 aa	Q8AYA2
PLA_2_ inhibitor	*B. jararaca*	331 aa	A0A481S6S6
PLA_2_ inhibitor	*B. jararaca*	332 aa	A0A481S7E6
PLA_2_ inhibitor	*B. jararacussu*	331 aa	A0A481S718
PLA_2_ inhibitor	*B. neuwiedi*	332 aa	A0A481S8C9
PLA_2_ inhibitor	*B. neuwiedi*	332 aa	A0A481S6U1
PLA_2_ inhibitor	*B. alternatus*	331 aa	A0A481S7I6
PLA_2_ inhibitor	*B. alternatus*	331 aa	A0A481S6U8
PLA_2_ inhibitor	*B. moojeni*	331 aa	A0A481S725

## Data Availability

No new data were generated in this study. All data discussed are publicly available from the cited literature and online databases such as UniProt, AlphaFold, PubMed, SciELO, and NCBI as properly referenced. Additional information or supporting materials are available from the corresponding author upon request.
